# Vulnerability, Disability, and Agency: Exploring Structures for Inclusive Decision-Making and Participation in a Responsive State

**DOI:** 10.1007/s11196-022-09946-x

**Published:** 2022-12-21

**Authors:** Maija Mustaniemi-Laakso, Hisayo Katsui, Mikaela Heikkilä

**Affiliations:** 1 Institute for Human Rights, Åbo Akademi University, Turku, Finland; 2 Faculty of Social Sciences, University of Helsinki, Helsinki, Finland; 3 Faculty of Social Sciences, Business and Economics, Institute for Human Rights, Åbo Akademi University, Turku, Finland

**Keywords:** Vulnerability, disability, human rights, agency, participation

## Abstract

By unpacking some of the dichotomies inherent in the concepts of vulnerability and disability, the article problematises some of the current legal approaches to disability in Finland. It argues that where used to single out population groups or individuals due to their embodied characteristics, the vulnerability paradigm can be seen to create binaries both among the persons with disabilities, and between the “vulnerable” persons with disabilities and the perception of a rational, self-standing and autonomous human being. To mitigate such binaries, the article explores an agency-centred discourse of vulnerability, one that recognises the co-existence of agency and vulnerability and sees agency as dynamic and responsive to the societal support structures that surround all of us. One of the central arguments of the article is that generalised approaches do, however, not suffice to make agency a reality for all persons with disabilities. Given the extensive diversity of intra-group variations between persons with disabilities, individualised solutions are needed for agency to be possible for all. To overcome objectification and de-agencification – and to enhance agency – this diversity of situations, needs and contexts of lived-in realities of individuals also needs to be expressly reflected in the legal language in addressing disability.

## Introduction

In discussing how a state should approach disability in welfare states such as Finland, the emphasis is often on the so-called universal design of societal structures in which all persons, regardless of their age, size or disability can fully participate without the adoption of numerous individualised measures. Such a universalistic approach to accommodating the diverse needs and the range of abilities and disabilities people have is in many ways important. Yet, where disability primarily is addressed through the universal design paradigm, the variety of the different lived-in realities and the role of many persons with disabilities as active agents in decision-making affecting them is typically not put in the focus. Where, on the other hand, individualised measures are adopted to “protect” or “help” persons with disabilities, the emphasis often is on the “vulnerability” of the persons with disabilities, or a care-giver–object of care paradigm, whereby the role of the persons with disabilities often is reduced to that of the protection-receiver. With this in mind, the article problematises some of the current legal approaches to disability in Finland, which often seem to be based on a binary approach, where some people are regarded as rational, self-standing and autonomous, whereas others (the “vulnerable”) often are approached using a rhetoric that portrays them as dependent and passive objects of protection. Today, this binary takes place not only between persons with and without disabilities but also among persons with disabilities. For instance, in Finland, persons with physical disabilities can utilise personal assistant services to materialise agency where needed, while many persons with intellectual disabilities are not legally allowed to access this service due to their “incapacity”.

The central question in the article is, therefore, how the law could recognise the diverse and heterogenous sources to vulnerability for persons with disabilities, without falling into the traps of objectification, stereotyping and de-agencification. To overcome the objectification inherent in many of the current approaches to vulnerability, the article argues that a more informed approach to vulnerability is needed in law to accommodate the variety of the different realities of persons with disabilities and the different measures that are needed for them to have their voices heard/represented in decision-making affecting them. In adopting such an approach, attention needs to be paid to the fact that persons with disabilities cannot be approached as a homogenous group. As such, the state, and humans alike, need to be attentive to the varying needs of the persons with disabilities. While some persons with disabilities act as independent and active agents, some persons with, for example, severe intellectual disabilities, may need significant support in realising their agency, or co-agency.

The article explores these questions by unpacking some of the dichotomies inherent in the concepts of vulnerability, disability, and dependence. It does so by first looking into the semiotics of the said concepts, moving then to explore the conceptualisation of agency of persons with disabilities and the different measures that are needed to overcome the hurdles that persons with disabilities face in exercising their agency. With the help of examples drawn from the Finnish system of disability services, the article points to structural impediments for the realisation of agency by persons with disabilities, showing that the discourses on disability are both reflected in and, arguably, construct the legal realities that the persons with disabilities live in. Finally, with a reference to the on-going renewal of the disability services legislation in Finland, some of the possible solutions to overcome the de-agencifying effects of legislation will be discussed.

## Identifying and Naming: The Universal and Particular Dimension of (Dis)Ability and Vulnerability

Persons with disabilities – some groups of persons with disabilities in particular – are often identified as vulnerable. Generally, this is done to attach attention to the enhanced obligations states have to attend to their particular needs for protection and support. Such obligations that states owe towards persons with disabilities as “vulnerable” have been highlighted in numerous cases of international human rights law. In *Fernandes de Oliveira v. Portugal*, the Grand Chamber of the European Court of Human Rights, for example, characterised the “mentally ill” as “particularly vulnerable” and noted that authorities should demonstrate special care in guaranteeing that the detention conditions “correspond to the person’s special needs resulting from his or her disability”. Likewise, in *Autism – Europe v. France*, the European Committee of Social Rights held with a reference to persons with autism that states “must be particularly mindful of the impact that their choices will have for groups with heightened vulnerabilities” [1:para. 53]. A similar idea of special protection is found in the 2006 UN Convention on the Rights of Persons with Disabilities (CRPD), which recognises the need for reasonable accommodation, that is, “necessary and appropriate modification and adjustments” in particular cases to ensure that persons with disabilities can enjoy their human rights at par with others [2:art. 2]. Overall, such special protection within human rights law both internationally and in many domestic legal systems is instrumental in reminding decision- and policy-makers of the fact that persons with disabilities are entitled to effective access to the same spectrum of rights as anyone else. At the same time, the vulnerability paradigm as a politico-social tool does not come without certain risks and pitfalls as regards, for example, selectivity and bias in terms of identifying special protection needs and vulnerability [3]. Such legal labels may in some contexts constitute gatekeepers to assistance and protection, whereby they may become a powerful means of governance [4].The use of vulnerability as a legally relevant benchmark may, as such, even lead to situations, where individuals feel the need to be categorised as vulnerable to gain access to certain forms of protection [5].

At the same time, the *language* of vulnerability and disability, and the stigmatisation and stereotyping that such concepts commonly entail, can be utterly disempowering and objectifying [6–8]. This is true particularly for persons with disabilities given that disability in many contexts continues to be approached in negative terms of fixing [9: p. 81] a “disadvantage” or a “deficit” [10], along the lines of the *medical approach* to disability seeing disability as a result of a person’s impairment [11]. When disability is defined or approached using terms with negative connotations – as in “suffer from epilepsy”, “multiple sclerosis victim” or “wheelchair-bound” – discourses can contribute to constructing negative attitudes about disability and thereby affect the policies that are undertaken [12:29]. The charity and welfare based discourses that we use in relation to disability, again, may lead to objectifying and marginalising persons with disabilities, thereby sustaining existing power structures and separating “us” from “them” [for a dicussion, see, 13]. This paradox between “the norm” and “deviance” is present also in the dichotomous and outdated vocabulary of able vs. *dis*abled, of which the semiotics traditionally entails a “signifying system”, a way of distinguishing individuals based on an embodied characteristic or capacity [on signifiers, see, 14:10].

Typically, the focus in the debates on the *relative dependence* of some persons with disabilities on the societal or other support structures has entailed that their participation in public policies has received less attention [15:26]. In many ways, persons with disabilities therefore are trapped into perceptions of vulnerability shaped by how the society responds to different exposures of dependency and autonomy [cf. 16]. These perceptions of vulnerability and the different degrees of self-sufficiency of individuals are often met with an imposed paternalistic approach by the society towards the “vulnerable”, coupled with an assumption of reduced or lacking agency of the individuals in question often also assuming that experts know what is in their best interest [10]. The resulting marginalised role in decision-making has “[…] serve[d] to reinforce stereotypical assumptions about the incapacity of persons with disabilities” [15:26]. As Fineman notes, “[t]hose who are not seen as sufficiently autonomous and independent actors are herded together in designated ‘vulnerable populations’ and are susceptible to monitoring, discipline, and supervision”, often with their agency withheld or taken away [16:84–85]. On the same note, Butler observes that “[o]nce groups are marked as ‘vulnerable’ within human rights discourse or legal regimes, those groups become reified as definitionally ‘vulnerable’, fixed in a political position of powerlessness and lack of agency” [17:24–25]. This can materialise in different ways. Traditionally, as noted by Yoshida and Shanouda, different practices have been used to attempt to silence the experiences of persons with disabilities [18]. This can, likewise, be a result of the stigma and the negative attitudes often attached to the labels of vulnerability and disability, especially as regards the label of intellectual disability, which guide decision-making about persons with disabilities [19]. The idea of deviance inherent in such labelling may also become a “self-fulfilling prophecy”, affecting the self-image of the labelled persons and eventually turning the label true [20].

Different vulnerability and disability theories have questioned these assumed binaries between “the vulnerable” and “the invulnerable”, and “the disabled” and “the able” [for a critical discussion, see, e.g., 21], arguing for a more holistic understanding of vulnerability, ability, dependence, and autonomy. For example, Fineman views dependence and vulnerability as something that we all encounter: “As embodied beings, we are all constantly vulnerable to events that might render us dependent” [16:86]. Therefore, whereas the connotation of vulnerability often is linked to individuals who in some regards depend on others, such as many persons with disabilities, she argues for a more universal take on both the concept of vulnerability and dependence, which both are seen as “inevitable” aspects of the human condition that we all encounter within our life course [16:86–87]. As such, vulnerability should be seen as an inherent and constant characteristic of all of our lives [22]. While Fineman opts for a changed understanding of what it means to be human (vulnerability and dependence instead of rationality and autonomy) and an understanding of vulnerability that underlines its universal character, some others rather challenge the vulnerable-invulnerable binary by noting that vulnerability is essentially a matter of degree. In this regard, De Beco [23:47], among others, referring to Wolff submits that “[…] a strict separation between the notions of dependency and of independence –​and accordingly those of disability and of ability –​does not make sense”, arguing that dependency, like ability, is never absolute in the sense that persons even with severe forms of impairments would lack any form of agency. Rather, he argues, persons’ agency is contextually dependent and varies depending on the circumstances that we find ourselves in. In other words, the difference between the “disabled” and “abled” is not so much a question of either or but rather one of degree [23:47].

Much in the same way, Mackenzie et al. note [24:7] that vulnerability is intrinsic to the human condition and arises from “our corporeality, our neediness, our dependence on others”, with some of it presenting itself constantly, other vulnerabilities depending on factors such as our health, age and disability. By distinguishing between different sources of vulnerability, they emphasise that human vulnerability essentially is caused by the interaction between humans and their environment, with *situational vulnerability* arising from or being exacerbated by personal, social, economic, or environmental factors. Such an understanding of vulnerability that stresses the interaction between the individual and his/her/their environment in essence aligns with the often-cited definition of disability in the CRPD. According to the convention, disability is an “evolving concept” and it “results from the interaction between persons with impairments and attitudinal and environmental barriers that hinders their full and effective participation in society on an equal basis with others” [2:preamble, para. 5]. In other words, disability is understood as relational, taking place between persons with disabilities and their environment, context, history, politics and other individuals, including persons without a disability. With this understanding, a gradual shift has taken place from a corrective or medical approach to persons with disabilities, focused on treatment, medication, and rehabilitation, towards a social or a human rights-based approach to disability, seeing disability as a universal characteristic of the human life and to increasingly focusing on mitigating and removing the societal and structural barriers that hinder the equal participation of persons with disabilities in society [25, 26]. This understanding of disability lies behind the focus on universal design in Article 2 of the CRPD, defined as “the design of products, environments, programmes and services to be usable by all people, to the greatest extent possible, without the need for adaptation or specialized design”. This approach is visible also in that the convention marks a shift in speaking of “persons with disabilities” instead of “the disabled”, reflecting the idea that disability arises from the interaction between persons and their environment and underlining that individuals therefore are not defined by disability. Such a move from the “identity-first” expressions to “person-first” terminology counteracts stereotyping and highlights the fact that “[d]isability is a part of life and of human diversity […]” [21, see also, e.g. 27].

However, when concrete decisions about access to special protection are made, a conceptualisation of vulnerability or disability that starts from the premise that everyone is equally vulnerable or abled is not always found helpful. That is why further criteria are often identified, for example, in assessing the eligibility of persons to disability services to meet their disability-related needs [28:10–11]. In practice, therefore, in many contexts the medical approach to defining disability continues to play a considerable role in assessing the needs and thus the eligibility of a person to disability services [28]. This is so even though the medical model is heavily criticised for ignoring the underlying social aspects of disability [11, 21]. For instance, in Finland, the embodied understanding of disability is currently reflected in Article 2 of the Finnish Act on Disability Services and Assistance (380/1987), which defines *a disabled person* as someone who *due to disability or illness* experiences particular and long-term difficulties with his/her/their normal life [29]. The definition does, as such, not capture the person-first approach of the CRPD underlining that disability does not define a person but arises from the persons’ interaction with their environment. This is visible, also, in the statement of the objectives of the act (Article 1), which include the prevention and abolition of impediments and hurdles caused by disability, reflecting the idea that the hurdles and impediments are caused by a person’s embodied condition. Access as a subjective right to some disability services, such as personal assistant service and transportation service, require the meeting of additional criteria, such as a severe disability or intensive and continuous support needs in one’s daily life [28]. In the Government’s proposal of October 2022 for a renewal of the law on disability services, a move away from diagnosis-based understandings of disability towards individual and contextual needs-based assessment in interaction with a person’s environment is suggested [30:1, 55, 120–121]. Diagnoses, however, seem to a certain degree remain as a suggested basis for assessing the legal base of services for old persons, with access to special protection under the law on disability services being available only for protection needs stemming from a disability that is not connected to aging [30:55, 158–161]. This has been criticised for accentuating the *cause* of, for example, a hearing disability as a threshold for access to disability services, instead of the actual individual need for such services [31].

In other words, while a shift is taking place towards viewing vulnerability as a universally shared characteristic, the identification of special needs in terms of service provision continues to entail some degree of essentialism and categorisation arising from the recognition of embodied or inherent vulnerability. As noted by de Beco, the dilemma is that “[h]ighlighting particular forms of embodiment may result in losing sight of vulnerability as a universal experience, but not doing so could lead to real needs being ignored” [23:81]. In our legal approaches to disability, we need, in other words, to be sensitive to both the need for conceptualising the needs for special protection, and the role of such concepts as sources of stigma and disempowerment. As lawyers, law-makers and researchers we should, therefore, be aware of the semiotics of the terms and discourses that we use, acknowledge their power, and understand the roles that the terminologies that we use may have in terms of creating meanings [cf. 32:v] and maintaining certain ideologies. Here, understanding language as an *interactive* art that is always understood in a *context* is key [cf. 33]. Considering this, it is important to recognize that categorisations and labels are more than mere linguistics. This means that we need to be “carefully attuned to the implicit biases and societal messages that pervade our ability to understand individuals, problems, and solutions” [34]. In everyday as well as in legal language, concepts gain meanings that have legal and other effects on the lives of persons with disabilities and can have a role in maintaining cultures of silencing and sustaining unequal power structures. After all, as Lazar notes, “[e]very act of signification through language and other forms of semiosis contributes to the reproduction and maintenance of social identities, relations and orders as well as to contesting and transforming them” [35:374]. As such, all discourses may contribute to promoting, but also to undermining the interests of specific individuals and groups, and can play a role in sustaining divisions between in-group and out-group representations [36:3]. In this way, the choices that we make in terms of the discourses that we (re)produce, mainstream, and cement in relation to disability all contribute to the social and legal construction of persons with disabilities as well as their needs, autonomy, and dependence. Such choices are key in building perceptions and attitudes about disability and any misconceptions that they relay may sustain negative self-perceptions of persons with disabilities, as well as institutional discrimination against them [12:29].

Binary usages of concepts, such as vulnerability/invulnerability and disability/ability, are often problematical, as such an understanding of the concepts fuels perceptions of deviance from normality and underscores the dominant narrative of the independent, self-sufficient and autonomous human being as the idealised norm [16:87–88. Also see, 37:327–331]. At the same time, the concepts of vulnerability and disability can also be sources to empowerment as advocacy tools and as vehicles in claiming rights. Who gets to define disability and vulnerability and their connotations is an instrumental question here, and one which gathers considerable social, political, and legal significance. In terms of self-identification, it should be up to each person to decide whether or not one identifies oneself disabled as disability is strongly attached to questions of identity [38:12]. The same applies to vulnerability. Such self-identification is reflected in the active disability communities that for example in Finland powerfully advocate for the rights and the substantive equality of persons with disabilities, and in which the disability identity often creates a basis for empowerment and advocacy [38:12, 39]. If used as a heuristic tool to unmask and to address contextual factors giving rise to vulnerabilities, such discourses can, in other words, have potential as a tool for resistance by the disability community itself against the prevailing power structures and ableism [cf. 40–43].

It also should be acknowledged, as de Beco notes, that “[t]here is in fact no term that refers to ability and disability in a non-binary manner” [23:83]. The same can, perhaps, be said about the notion of vulnerability, which, at least to an extent, has come to replace related concepts such as “marginalisation” and “disadvantage”, which, just like vulnerability, carry their own connotations of stigma and stereotypes. Simply replacing one concept with another one, therefore, does not seem to be sufficient to solve the issues of stigmatisation, de-agencification and stereotyping. Rather, it is argued in this article, the essential should be the content that the concepts are given through transposing them to a legal context and, centrally, the ways in which the different understandings are supported by the underlying legal structures and their implementation. After all, non-binary conceptualisation as such is not very helpful if binaries are created through legal solutions that lead to paternalistic or objectifying outcomes. Below, in Sects. 4 and 5 of this article, this question will be addressed in relation to some areas of disability legislation in Finland with the aim to understand how the legal structures can both facilitate and set hurdles for the co-existence of vulnerability, special protection, and agency.

This perspective to defining and addressing disability is central in adopting a human rights-based approach to disability, where persons with disabilities as rights-holders are seen as active subjects of their own rights, not as objects of welfare and protection, and are expected to be given a voice in both defining disability and in all decisions affecting them. When discussing legal approaches to disability, and to identifying persons with disabilities, great care is, therefore, to be taken not to lose sight of the fact that vulnerability and disability should not rip individuals of their right to be in – or take – control over their own lives, and the possibilities and openings that this entails. The conceptualisations that we choose to use, and the ways we use them, as well as the legal and other societal structures that we create based on such conceptualisations, should, in other words, not downplay the *agency* of the human being, in particular of persons with disabilities, who traditionally have met challenges in having their participation rights realised [44:para. 1, 45]. To this end, we need to understand the different roles and meanings of agency in the context of disability, and what needs to be done for it to cement its place within the vulnerability (or special protection) rhetoric.

## What is Agency and What Is It Not? And Why Does It Matter?

As a concept, agency tends to take on different meanings depending on the context. Often markers such as power, resistance, autonomy, and possibility to affect one’s own life are attached to the term [46, 47]. A generalised conceptualisation describes agency as “the socioculturally mediated capacity to act” [47:112]. Three elements of this definition should be highlighted for the purposes of this article. First, agency is seen as a “capacity to act”, with the “act” referring to an active handling not merely a decision to refrain from a given conduct. Second, it should be noted that “capacity”, as will be discussed more in detail below, can take different forms and can also be exercised through proxies. Third, that such capacity is “socioculturally mediated” entails that agency, much like resilience, is highly dependent on and fluctuates with the changing contexts and the enabling factors surrounding an individual.

In this way, vulnerability and agency interact in different ways [cf. 48:639]. Central in this regard is to recognise that, as noted above, agency and vulnerability are not mutually exclusive. The tendency to think of these two in dichotomous terms has been observed, for example, with regard to women, who often tend to be portrayed in law and policy *either* as vulnerable victims (such as victims of domestic violence or victims of trafficking) *or* as non-vulnerable agents (for example in rape cases where a woman is found to have presented sexual agency) [49:36–37, 50]. Brown notes, in a similar vein, that in policies relating to the care and control of young people, the nexus between vulnerability and transgressive behaviour is rarely recognized and young people often are dichotomised into “vulnerable victims” or “dangerous wrong-doers” [51:371]. Based on informant interviews, she notes that “young people’s entitlement to support was most secure where they ‘performed’ vulnerability through displaying ‘conformist’ behaviours.” [51:379–380]. Such dichotomies, Mahoney notes, reflect “the ways agency and victimization are socially defined in relation to each other”, with victimization entailing a “one-way exercise of power”, whereas agency is understood as entailing “freedom from victimization” [52:62]. Much like in the case of persons with disabilities, the agency of women, for example, is often portrayed through a “dichotomous view of female vulnerability/autonomy” that is “favoured over a more nuanced understanding of the culture- and situation-bound aspects of agency” [49:24]. Such an understanding fails to recognise the socio-culturally mediated nature of agency, and the fact that agency is entrenched in and facilitated by the different social structures and dependencies that we find ourselves in [cf. 48:639].

Individuals’ agency, just like resilience, should, in other words, not be seen as something static, but rather as something that fluctuates and develops with the contexts that individuals find themselves in. Such an approach finds support in vulnerability theories. A central element in Fineman’s approach to vulnerability, for example, is the idea of a responsive state, which contributes to the resilience of individuals by providing tools for and by removing obstacles for the enjoyment of rights [53]. According to this model, resilience, that is, an individual’s “means and ability to recover from harm, setbacks, and the misfortunes” [22:146], is socially produced and is as a result sustained, enhanced and hampered by institutional and other arrangements and relationships in a society. Such resilience-building as a central duty of the responsive state also forms the backbone of the human rights-based approach to disability as expressed in the CRPD and its central tenets of universal design and reasonable accommodation [54]. On a similar note regarding agency, with a so-called ecological understanding of the concept, as Biesta and Tedder note, our focus when looking at agency is shifted from something that is *possessed* to something that can be *achieved* through the active engagement of individuals with their environment [55]. Cooke observes, correspondingly, that while agency is entrenched in culture, individuals are “complex and contradicting actors” and as such they move in between, and “engage with and are influenced by the different social organisations differently” [49:29].

Participation, undoubtedly, is an element of agency, and the definitions of the two terms overlap to some extent. As a “multidimensional concept”, Gesser et al. note, participation can cover “a spectrum of aspects – from the practice of activism to the constitution of subjectivity in disabled people” [56:1772]. Generally, participation is interpreted as taking decisions affecting oneself and the possibility to be involved in decisions about, for example, societal matters [see further, 57]. The CRPD recognises the right to participation for persons with disabilities in several articles, including Article 3, which recognises their full and effective participation and inclusion as a general principle as a general principle of the convention. Articles 29 and 30, again, guarantee the rights to participation in political, public life and cultural life, as well as in recreation, leisure, and sport. Article 23 (1) of the Convention on the Rights of the Child further determines that all children with disabilities are to enjoy a life in dignity in conditions which ensure self-reliance and facilitate active participation in the community.

Importantly, the right to participation and agency for persons with disabilities does not equal mere rhetoric, pseudo, or one-off, involvement by persons with disabilities but requires that subjects have meaningful ways to influence the decisions that are made [58:131–132]. To be meaningful, participation needs to be effective, that is, it must have an effect on the decision-making in that the views that are expressed are given due account to and that the participants are given a meaningful possibility to form informed views based on accessible and available information. This understanding of agency is found also in the General Principles of the CRPD that guide the interpretation and the implementation of the convention. Among these general principles are included individual autonomy comprising the freedom to make one’s own choices, as well as full and effective participation, inclusion in society, equality of opportunity, and accessibility. Likewise, the Preamble to the convention underlines the “valued existing and potential contributions made by persons with disabilities to the overall well-being and diversity of their communities”, as well as “the promotion of the full enjoyment by persons with disabilities of their human rights and fundamental freedoms and of full participation by persons with disabilities” that “will result in their enhanced sense of belonging and in significant advances in the human, social and economic development of society and the eradication of poverty”. For these reasons, it is held in the Preamble, “persons with disabilities should have the opportunity to be actively involved in decision-making processes about policies and programmes, including those directly concerning them”. Overall, one could, in other words, argue that the convention encapsulates an agency-based approach to disability, arising from the fact that it views persons with disabilities not only as subjects of their own rights but also as active actors in claiming them. Thereby the convention captures the very essence of agency that transforms the image of persons with disabilities from objects of charity to active agents of their own rights.

This is important as agency and participation are key elements of – and entry points to – individuals’ empowerment and rights and they function as preconditions for individuals’ capability to exercise choices, activism, and power over their own lives. For persons with disabilities, as for everyone, they are part and parcel of the right to self-determination [see e.g., 59], which, together with personal autonomy, are essential to, inter alia, independent living [44:para. 16]. Participation is also found to be the most effective way of ensuring individuals to “experience the society – or its sub-systems – as their own” [60:42]. As recognised in the Preamble to the CRPD, agency of persons with disabilities additionally serves an instrumental aim in that informed decision-making on matters affecting them requires meaningful participation by persons with disabilities themselves to enable decisions reflecting lived-in realities and needs. Notably, meaningful participation by persons with disabilities is key to unmasking ableism, and “abled privilege” in how we portray disability through the rhetoric and language that we use in law- and policy-making, as well as to point to the potential of “empowering attitudes and values” [61:15, 34]. This is, in many ways, the rationale of the different disability movements and theories that aim at “taking control” over the meanings of disability and to deconstruct the collective structures of power and attitudes that prevent persons with disabilities from making choices about their lives [61:33]. Below, a look will be taken at what such impediments to the agency of individuals with disabilities may look like in practice, and what could be done to overcome them.

## Hurdles to Inclusion of Persons with Disabilities as Active Agents of Their Own Rights in a Responsive State

Hurdles for participation and agency can take many forms, including the fact that persons with disabilities are not systematically and meaningfully involved in decision-making affecting them [e.g. 62]. In Finland, challenges are reported as regards the participation of persons with disabilities in decisions affecting their services, in terms of involving them in decision-making and in taking their views into account, leading to situations where their participation is only formally realised [30:49]. Such lack for room for self-determination and agency is paramount, for instance, in numerous examples in the deinstitutionalisation process of persons with intellectual disabilities, even to the point that such lack of participation can be referred to as an institutionalised element of the operational culture during the process of the deinstitutionalisation and even after physical relocation [63]. This is reflected in research with participants with various backgrounds reporting of an evident lack of choice when relocation takes place for persons with intellectual disabilities [63]. A representative of a local disabled persons’ organisation accounts, for example, that “[l]ack of choice means that it is informed always only where is the place [to move into] now. [The way how the city promises the place is] more or less take it or leave it. In those cases they don’t listen to you. So when the city makes some decision, you just have to live with it” [64:41]. The agency to choose one’s living arrangements may also be limited due to lack of support, support being restricted to certain living arrangements, their lack of accessible universal design or lack of arrangements for independent living in the community [44:para. 1].

As regards the accessibility and availability of support sustaining the agency of persons for disabilities, it should as well be noted that for the persons with disabilities the binarisation between the invulnerable and the more or less vulnerable takes place not only as compared to persons without disabilities but also among persons with disabilities. In Finland, for example, while more focus has lately been put on the individual assessment of service needs, for persons with intellectual disabilities the differentiated service structure in the Act on Intellectual Disabilities (519/1977) with group- and institution-based service forms has in practice largely defined the services available for them [30:46]. This has created hurdles for the exercise of agency for persons with intellectual disabilities and may have contributed to their marginalisation [30:46]. Also, subjective rights for personal assistance are in Finland granted to persons who are considered to have sufficient capacity/resources to exercise self-determination rights to define the content and the ways of operationalising such assistance [29:8(c)§, 65, 66]. Accordingly, for example, some persons with severe cognitive disabilities and autism have been left out from the purview of such services [67:53]. As the assistance is intended as a vehicle to independent living and self-determination, and thereby to reinforcing the capacity of the users to make decisions concerning their daily lives, it shall, in other words, be “person-directed/user-led” [44:para. 16(d)]. Paradoxically, as such, the availability of personal assistant services for supported agency is, in other words, to a certain degree conditioned on previously existing agency.

The reasons behind the inclusion of this so-called capacity (or resource) criterion, introduced in the Finnish legal system through the Act on Disability Services and Assistance (380/1987) in 2009, draw on the rationale that for the assisted persons to be able to meaningfully benefit from the assistance, they need to be able to express their wishes concerning such assistance [67]. Such an approach is reflected in the General Comment No. 5 to the CRPD concerning independent living, according to which personal assistance service “must be controlled by the person with disability” either as a contractor or as an employer and must be self-managed through a self-selected degree of control over service delivery according to the own preferences by persons with disabilities [44:paras 16(d)(ii) and (iv)]. The capacity criterion was largely maintained in a February 2022 draft proposal for the renewal of disability laws in the country , with special support (*erityinen tuki*) suggested as a new alternative service form to meet the needs of persons with intellectual disabilities [67]. The binaries that this was found to create among persons with disabilities have given rise to heated discussion within the disability community, with organisations supporting persons with intellectual disabilities typically advocating for the inclusion of persons with intellectual disabilities as service users of personal assistant services in the forthcoming law, while organisations of persons with physical disabilities generally insist on the importance of the capacity requirement as an entry criterion to be eligible for the personal assistant services [e.g. 68:21–33].

The recent decision by the UN Committee on the Rights of Persons with Disabilities concerning a claim against Finland on the availability of personal assistance to a person with a physical and intellectual disability [69] is, therefore, as noted elsewhere principally significant [70, 71]. The decision clearly indicates that the provisions in the current Act on Disability Services and Assistance do not fully meet the requirements of the CRPD and should be interpreted in a way that does not prevent the realisation of the rights of persons who need support in determining the content and the modalities of the required assistance. In its findings the committee submits that Finland has declined the request by the author for in-home personal assistance “based on the grounds that he would be unable to choose, a seemingly ableist argument contravening the human rights model of disability” [69:para. 9(3)]. The committee therefore finds a violation of Article 5 (1) and (2) read alone and in conjunction with Article 19 of the convention given that the refusal constituted indirect discrimination as it “had the effect of impairing or nullifying the author’s enjoyment and exercise of the right of living independently and being included in the community on an equal basis with others” [69:para. 9(8)]. The committee bases its finding on the fact the State Party had failed to present the applicant with an alternative arrangement under Article 19 (b) of the convention, as a result of which the applicant had been deprived of “access to a practical option that could support his living and inclusion in the community”, and thereby his rights under 19 (b) of the convention had been violated [69:paras 9(3) and 9(8)].

Another area where practical and structural barriers – including at the level of discourse and language – create obstacles to agency for persons with disabilities is employment. While the CRPD in its Article 27 lays out specific guarantees for the right of persons with disabilities for work and employment on an equal basis with others, these rights often remain inefficiently realised. For Finland, for example, the right to work has been found to be the least realised among the rights of persons with disabilities [72:52], with a considerably low number of persons with disabilities participating in working life, despite educational and rehabilitative efforts over the years [73:28]. Reaffirming the finding of an earlier report [74], a Ministry of Labour commissioned study on the structural barriers to employment faced by persons with disabilities indicates that the root cause of the current employment situation of persons with disabilities lies in the lack of political commitment [75:11]. The employment of persons with disabilities is considered as important in speech but is not sufficiently or effectively implemented in practice [75:39].

Among the many kinds of disability services, personal assistants and transportation services are highlighted as the most essential and often as preconditions for employment [75:19]. Nevertheless, the survey study of the Finnish Disability Forum found that for many respondents these disability services are not sufficient or do not sufficiently take into account personal needs [76:14–20]. Municipalities are, for example, to provide transportation services for study and work as well as at least 18 times for other activities per month [77:6§]. In practice, however, this is sometimes interpreted as the upper limit of the transport services to be provided as a subjective right, which may constrain the opportunities of persons with disabilities to participate, for example, in employment interviews [75:21–22]. Concerns have also been expressed regarding the lack of individualised accommodation of needs of persons with disabilities in the planning of transport services, which puts persons with disabilities in internally unequal positions and endangers the possibility of some persons with disabilities to engage on an equal footing with others in private, work and study life [78]. Also lack of knowledge – for persons with disabilities, employers and authorities – and geographical discrepancies in the service and support provision may function as hinders to this end [75]. Problems of inequality among municipalities are reported, for example, in relation to the realisation of the subjective right of persons with disabilities to personal assistance services. Such concerns relate not only to different interpretations of the decisions on services but also to different salary levels of personal assistants have been observed among different municipalities, Kyröläinen notes [75:26]. In terms of employment opportunities, the delays in receiving a decision on personal assistant services that should be given within three months may prevent the materialisation of employment opportunities as the need for personal assistant services may arise sooner [75:24].

As a result, structural and legal barriers to employment among persons with disabilities persist, including obstacles related to the inflexibility of personal assistant and transportation services for job seekers, the benefit system, recruitment and working life [75]. Indicative of this is that in Finland, a considerably low number of persons with intellectual disabilities are employed in the open labour market [79, 80]. Instead, persons with intellectual disabilities often are engaged on rehabilitative work activities at day activity and work centres (*päivätoiminta)* or at ordinary workplaces (*avotyö*). Such rehabilitative work is not reimbursed in salary but persons involved in rehabilitative work can receive 0–12 EUR per day, on average 5 EUR per day [81], in incentive pay for sheltered work. Persons in rehabilitative work are, in other words, not in an employment relationship with their place of work but conduct work as clients of social welfare, which means that such persons are not covered by labour rights related to, for example, protection against dismissal or annual leaves, something that persons with disabilities and their organisations are deeply concerned about [82, 83]. Notably, rehabilitative work does not either seem to function as an effective stepping board to open market employment for persons with disabilities [82, 84:21].

For persons with disabilities working in the open market employment the current benefit system is not encouraging for promoting the employment of persons with disabilities. At present, the allowance for those with work incapacity due to illness and disability is up to 855,48 EUR per month when the employment-related income does not exceed this [85], a condition which restricts persons with disabilities from working in higher salaried jobs [75:31]. Entrepreneurs, again, are not permitted to receive work incapacity allowance and a start-up fund at the same time, which limits their opportunity to found a company [75:57–58]. Overall, the current benefit system is, in other words, not encouraging for promoting the employment or self-employment of persons with disabilities. This is reflected also in the in-work training opportunities for persons with disabilities. Inexperienced persons with disabilities can be trained, during which employers receive training compensation from the government, while the employee with disability receives a salary. However, this training support is hard for the employer to obtain at the same time as work incapacity allowance, which contributes to the limited number of traineeships available to persons with disabilities that also negatively affects their competitiveness in recruitment in the open labour market [75:34, 48].

These structural and legal barriers indicate that in relation to work, persons with disabilities are not being conceptualised as active agents and employees but as objects of social protection. As a result, many persons with disabilities are dissatisfied with their employment services. Many of them are being told that they are not targeted customers as they receive a work incapacity allowance, even though they want to work. In fact, many persons with disabilities, as well as employment department staff members, do not know that they can also use employment services [75:37, 86:23–24]. From the perspective of agency, this dichotomous semiotics between an employee and a person who carries out work as rehabilitation (or as a “hobby”) is telling. The juxtaposition is reflected also at the level of legislation, where the rehabilitative work activities are covered as a *service* or *support* in the Social Welfare Act (2014/1301) [87:17§] or the Act on Intellectual Disabilities (519/1977) [88:2§], while *employment* within the open labour market is regulated through the acts on general labour legislation. Such a dichotomous approach has been questioned and it has been held that the guiding factor in defining in between an employment contract and work activity not covered by an employment contract should be the content of work, not the health of the participants or their basis of living [89]. Yet, the assumption of persons with disabilities as *external* to the working life and the parallels drawn between disability and invalidity for work, in other words, still largely persist [73:28], maintaining unequal power structures in working life. One underlying cause to this is that the general structures of employment and work, including legislation, have been developed with the “abled”, not the “disabled” workers in mind, which often means that such structures do not sufficiently accommodate the needs of the persons with disabilities in the working life, or in combining work with studies or family life [73:28–29], thereby cementing structures of ableism in the working life.

Another source to such de-agencification may be found in the strong focus on the protection/care-receiving paradigm attached to persons with disabilities. This has been highlighted, for example, in the global COVID-19 crisis. As noted by Brennan, “[p]olicymakers at many levels appear to have reverted to treating persons with disabilities as objects of care or control, undermining many of the gains of recent years to enhance citizenship, rights, and inclusion” [90:7]. In Finland, this was visible, for example, when restrictions were put in place under the COVID-19 pandemic for residents with disabilities in group homes and institutions to restrict the income of visitors, including family members with a view to protect the health of the residents [91:5–6]. Such measures focusing exclusively on the protection against the COVID-19 disease were subsequently found to be in contradiction with the law by the Supreme Administrative Court of Finland due to their effects on the private and family life [92]. Likewise, the Deputy Chancellor of Justice emphasised in April 2022 that when prohibitions of visits are adopted they must have a clearly pronounced legal basis (individual quarantine decisions adopted based on the Communicable Diseases Act), as such prohibitions infringe upon the fundamental and human rights of the residents [93]. From the perspective of individuals’ agency, it is particularly problematic that the lack of clarity concerning the legal basis of the restrictions, among other things, contributed to a situation where it was unclear to the residents where they could bring a complaint concerning the restrictions [94]. This was compounded by the fact that, as a legal question, the situation was found in the intersection of the Communicable Diseases Act (1227/2016) and social services legislation, of which the former largely is based on the idea of “patienthood”, whereas many of the situations faced with persons with disabilities usually take place within the field of social services [94]. Concerns are expressed that this shift during the pandemic measures towards considering the situation of persons with disabilities in terms of patienthood may have more permanent effects on how persons with disabilities are viewed, that is, as objects of regulation rather than as subjects of rights [94].

## Individualised Solutions for Accommodated/Enabled Agency

The above examples highlight the fact that protective measures may in some situations create hurdles to meaningful participation in society, albeit adopted with good intentions. To remove such hurdles and to instate the role of agency in the debates on vulnerability and persons with disabilities, due attention needs therefore to be attached to the societal and institutional structures, such as the law, as sources to “de-agencification”. Consequently, in our efforts to enhance the empowerment and agency of individuals, increasing attention should be paid to removing structures that create or sustain de-agencification, or “unfreedoms” which, as put by Sen, “[…] leave people with little choice and little opportunity of exercising their reasoned agency” [95:xii]. Following the taxonomy of vulnerability proposed by Mackenzie et al. this would mean addressing policies and structures that, even where meant as benevolent, generate so-called *pathogenic vulnerability* through stereotyping and “morally dysfunctional or abusive interpersonal and social relationships and sociopolitical oppression or injustice”, undermining autonomy or exacerbating the “sense of powerlessness” [24:9]. As they note, the idea of self-determined agency by Miller [96], and her notion of non-paternalism that nurtures such self-determined agency, aligns with this idea, critiquing the “forms of care that undermine agency” [24:12–13]. Thus, as “[…] being vulnerable can engender a troubling sense of powerlessness, loss of control, or loss of agency”, measures addressing vulnerability should aim “[…] to enable or restore, wherever possible and to the greatest extent possible, the autonomy of the affected persons or groups” [24:9].

In addressing disability, we need, therefore, to be mindful that the vulnerability discourse may both lend itself to justifying paternalistic approaches that create pathogenic vulnerability, and poses multifaceted normative questions as to the nature and role of autonomy as a factor in defining and facilitating agency. This brings us back to the sociorelational model of universal social vulnerability discussed above, challenging the idea of deviancy of the vulnerability of the persons with disabilities, and shifting the attention to contingent factors exacerbating and mitigating vulnerability. In the case of persons with disabilities, this would entail, among other things, removing hurdles to effective participation, both at the level of legislation and in practice. This presupposes a turn away from a patronising approach to the vulnerability of persons with disabilities towards a more empowering and participative one. It should also be noted in this regard that the essentialism inherent in stereotyping can be regarded as “both a cause and manifestation of the structural disadvantage and discrimination of certain groups of people” [97:708], indicating that the currently widely used group-based approach to vulnerability should be abandoned for a more individualised assessment of vulnerability. Such a requirement is inherent also in the praxis of the European Court of Human Rights that has held blanket responses to persons with disabilities as unacceptable for example in *Alajos Kiss v. Hungary*, arguing that “the treatment as a single class of those with intellectual or mental disabilities is a questionable classification, and the curtailment of their rights must be subject to strict scrutiny” [98:paras 39–44].

In Finland, the current overhaul of disability service legislation presents an opportunity for a move towards such a more individualised assessment of protection and empowerment needs. The aim of the overhaul is to put persons with disabilities more in the driver’s seat as regards the implementation of their rights, and portraying them as agents in that regard drawing on the latest Government programme [99] that contains a number of action points promoting the participation and inclusion of people with disabilities [100:4]. This aspiration is expressly reflected in the semiotics of the recent Government proposal of October 2022 for the renewal of the disability service legislation, which largely encapsulates an *agency-driven* approach to addressing disability. This is visible in how the proposal states as its “main aim” the enhancement of the equality and agency of persons with disabilities in the society, as well as supporting their right to self-determination and independence [30:53]. An *additional* aim is stated to be the realisation of their rights to sufficient social services and care, and complementing the social welfare legislation with a special law that takes into account the special needs of persons with disabilities [30:53]. Moreover, through different services and an individualised assessment of service needs, the proposal seeks to remove sources to inequality for and among persons with disabilities, support the possibilities of persons with disabilities for interaction and for making decisions over their own lives, and to enable maximising the use of their own resources [30:54, 56]. In removing the hurdles for such aims, individualised special services are needed, the proposal notes, generalised measures are not sufficient [30:153]. Notably, services are in the proposal suggested to be defined as *necessary* where they are required to uphold a life with dignity, including where such services are needed for the realisation of person’s agency [30:55]. As such, where the criteria set out in the proposed law are fulfilled, persons with disabilities regardless of the form of their disability would have a subjective right to services that facilitate their agency [30:57].

To that end, in its proposal for the law of disability services, the government suggests complementing the selection of special services with new services for special support for participation (*erityinen osallisuuden tuki*), and for supported decision-making (*tuettu päätöksenteko*). The special support for participation is proposed as a service form for those persons with disabilities who need particular support for realising their agency, while supported decision-making is meant to provide support for persons who need help in building and expressing their will [30:58–59]. The suggested service forms are a response to the criticism on the so-called capacity criterion for access to personal assistance that were expressed, as discussed above, for example, in the consultation process concerning the draft proposal of February 2022. The current proposal retains the debated capacity criterion as an entry condition for access to personal assistance but in a somewhat less demanding format, that is, for a person to be eligible for such a service he/she/they must be able to express his/her/their will regarding its content (but not its implementation) and such a will can be expressed either independently or with support. The suggested introduction of alternative service forms for supported agency can, also, be said to reflect a step towards following the recommendations of the Committee on the Rights of Persons with Disabilities.

According to the committee, the capacity criterion linked to personal assistance does not necessarily preclude agency where alternative forms of support are provided to enable the achievement of self-determination and independence as set forth in Article 19 of the CRPD for persons with disabilities to live independently and to be included in the community. While persons with disabilities shall remain “at the centre of the decisions concerning the assistance”, such control can, according to the committee “be exercised through supported decision-making” [44:para. 16(d)(iv)]. This means that “[w]hat counts is not the ability to decide by oneself only what is valuable but the real opportunity to rely on others in order to make such decisions” [23:48]. Therefore, the committee recommended Finland to ensure that “legislation does not have the purpose or effect of impairing or nullifying the recognition, enjoyment or exercise of any right for persons with intellectual disabilities on an equal basis with persons with other types of disabilities when seeking to access personal assistance”, in particular that “the resource-criteria requirement based on the beneficiary’s ‘ability’ to determine the content and modalities of the required assistance is not an obstacle to the independent living of persons who require support in decision-making” [69:para. 10(b-c)]. The committee’s decision entails, as Rautiainen and Nieminen [70] note, that individuals have the right to make – and be enabled to make – independent choices about their lives to a greater degree than has conventionally been thought in the Finnish disability service thinking. As such, they submit, the legislation should always effectively guarantee meaningful self-determination, which requires that the individual needs for support are sufficiently recognised.

Supported decision-making should, in other words, not be seen as the opposite of self-determination, but as a tool enabling self-determination [101]. Such supported decision-making should be carefully distinguished, and marks a shift away, from substitute decision-making. Substitute decision-making that takes place, for example, through plenary or partial guardianship or judicial interdiction generally entails that legal capacity is removed from a person and that decisions are made based on “what is believed to be in the objective ‘best interests’ of the person concerned” [102:para. 27]. Supported decision-making, again, shall give primacy to the will and preferences of the person himself/herself/themselves without overregulating the lives of persons with disabilities, and respect human rights norms, including a person’s right to autonomy and to right to choose one’s place of living [102:para. 29]. The Committee on the Rights of Persons with Disabilities has repeatedly underlined that the states parties have an obligation to review their legislation to abolish structures for substitute decision-making, replacing them with and developing of systems for supported decision-making [102:paras 3, 26 and 28]. Supported decision-making shall be available to all, independent of, for example, a person’s mode of communication, and, notably, of a person’s level of support needs, which shall – “especially where these are high” – not constitute a barrier to access to support in decision-making [102:para. 29].

The rehaul of the legislation on disability services presents, as said, an important opening for enhancing the agency of persons with disabilities. At the same time, for such an opening to materialise into effective support forms for persons needing special support for their agency and decision-making, care needs to be taken that the services for co-agency and supported agency both formally and in practice meet the needs of their users at an equal level to others. If introduced through the renewal, it should therefore be made sure that the suggested special service forms adequately ensure agency on an equal footing with other persons with disabilities for persons who fall beyond the capacity criterion [e.g. 103]. Considering this, concerns have been expressed, for example, as regards the uneven level of service available under the special support for participation as compared to the support available through personal assistance. It is regretted in this regard, for example, that the provisions on supported decision-making are suggested to enter into force with a two-year delay as compared to the other provisions of the renewed legislation that are foreseen to enter into force in the beginning of 2023 [30:226]. The availability of special support for participation is also proposed to be subject to a transition period. This means that during a three-year transition period, support under the special support for participation would be available at the minimum for ten hours, and after that for twenty hours per month at the minimum [30:227]. Overall, criticism has been expressed concerning the fact that the proposed minimum amount of service available under the special support for participation would be lower than the 30 h per month available for recreational activities through personal assistance [104]. This would mean that those persons with disabilities who need most support for their agency in their free time would be eligible for least support, leaving them in unequal situations in terms of supported decision-making and agency in relation to other persons with disabilities and in general [104]. As regards supported decision-making, attention should be paid, as well, to the fact that the government proposal limits such support to *significant* decisions concerning one’s own life, such as the choice of study place or transfer or residence to another municipality, consequently leaving many smaller and more mundane, yet important decisions in a person’s life outside of such support. In such situations the realisation of a person’s self-determination would thus be dependent, as the government’s proposal states, on other services, such as personal assistance, support for housing, special support for participation, or the day and work related activities (*päivätoiminta*) organised for persons with disabilities [30:120, 197–198].

Considering this, it is worth noting that the implementation of the said support forms for agency is met with the current post-COVID budgetary situation as well as the somewhat altered prioritisations in terms of spending [100:4], which raises concerns about the actual implementation of such support. This is especially so as the forthcoming disability law is being planned in an essentially “budget neutral manner”, redirecting existing resources [105]. Worries have, as well, been raised by persons with disabilities about austerity measures linked to the ongoing reform of health and social services (SOTE reform) [106:21, 107]. Such worries can be seen as reasonable, considering that also the government proposal for the new legislation notes that, in practice, the level of services will be guided by the budgets and the decision-making at the regional government level [30:119]. Concern in this regard is being expressed regarding the procurement law passed in 2016, by which the decision-making concerning public sector procurement has been more strongly affected by market principles. The rampant market-oriented practices of disability service providers as well as their relationships with the municipalities that purchase their services were questioned in 2018 in a campaign by disability organisations called “Not for sale!” (*Ei myytävänä!* in Finnish) [108], which collected as many as 72,059 signatures and went to the Parliament for discussion. The campaign’s main criticism was directed against the recent trend of public sector decisions on disability services, including housing services for persons with intellectual disabilities where market-oriented competition quite frequently resulted in discontinuation and/or deterioration of disability services for many persons with disabilities [63]. With this in mind, for the suggested changes and the new service forms to be effective and for them to meaningfully support agency in practice, the adequacy of the resources reserved for such reforms should be carefully monitored and adjusted where needed [also see 104].

It is also important to recognise the role of service fees and customer charges, even small ones, as possible hurdles to active participation for persons with disabilities. In this regard it is noted that in the recent government proposal for disability service law, the fees for transportation to different services, such as day and work related activities (*päivätoiminta*), special support for participation, and supported decision-making, are suggested to become subject to a retention [30:115]. The resulting rise in expenses, if realised, is estimated to be a considerable impediment for some persons with disabilities [104]. Taking into account that these service forms are suggested, as described above, to form some of the “fall-back solutions” for persons not fulfilling the capacity criterion for personal assistance, it would be important to ensure that they are *de facto* accessible to those who need them for their agency to be realised.

Beside the rehaul of the disability service legislation, different scenarios are being discussed in Finland in terms of enhancing the employment opportunities for persons with disabilities, with employment quotas and the so-called Työkanava (“work channel”) model as the most prominent alternatives. In 2022, a state-owned special assignment company called Työkanava following experiences in Sweden was introduced for the employment of “people with impaired work capacity”, seeking to facilitate their inclusion in the open labour market through supporting their employment capabilities [109]. The model is presented as a “last resort” option and initially seeks to annually employ some hundred, and later some 1,000 persons, “in the most difficult labour market position” [109]. The aim as such serves an equalising purpose and from the perspective of agency it is significant that when performing work through Työkanava, persons with disabilities do so under a contractual employment relationship [110], not as customers of social welfare, which underscores their belonging to the active workforce as employees. However, the experiences in Sweden of the Samhall system indicate that the model has not been fully operational in enhancing the employment of those in most need of support for their employment [111]. It can also be criticised for creating another layer of specialised space for persons with disabilities, underlining the idea of disability as a distinguishing factor in employment.

The quota system that is in use in many countries has not been the selected option in Finland, but was recently brought up by the Minister for Social Affairs and Health as one alternative to enhancing the employment of persons with disabilities [112]. Within the disability community, the suggestion was received with mixed feelings. While some are more positive [113], others take a more reserved view and call for further assessments [114, 115]. The quota system does present possibilities in raising the low employment rate for certain groups of persons with disabilities by setting a minimum percentage for the employment of persons with disabilities to be attained. The challenges are, however, many. For example, collecting information of disability is sensitive, and in many countries, such as Finland, the possibility to do so is limited due to reasons of data protection. The quotas also often require persons to identify themselves as persons with disabilities, which may, again, contribute to accentuating their disability, differentiating them from the “other” employees and overshadowing their abilities and skills as employees [for a discussion, see, e.g., 116, 117]. While such disclosure, on the other hand, has been found to also have empowering effects in terms of awareness of one’s legal rights in terms of employment [118], in practice the proof that the quota systems would considerably improve the employment chances for persons with disabilities is still limited [118–120]. Research indicates also that persons with disabilities are “unequal in their capacity to make strategic use of the disability quota system” [118], which may leave some persons with disabilities further behind and marginalised in society.

Overall, therefore, one conclusion that can be drawn from the discussions concerning the different accommodations for the employment of persons with disabilities is that one solution does not accommodate all needs within the disability community. This finding finds support in research that indicates that successful integration of persons with disabilities in the labour market requires “a balanced setting of sanctions, incentives and support, which complement each other” [119]. Importantly, as well, disability inclusion should be understood well enough for the employment of persons with disabilities to have added value and not become an imposed requirement perceived in negative terms [116]. This means, in essence, that an attitude change towards the employment of persons with disabilities is central for any model to work. As noted by the Finnish Ombudsman for Non-Discrimination, a paradigm shift will be necessary to this end, one that perceives persons with disabilities as independent subjects of rights, not as objects of care [73:29]. To a certain degree, globally, such a change in attitudes is reportedly already taking place, with organisations becoming aware that the employment of persons with disabilities is in their interest, given, for example, the shortage of skilled workers and the aging labour force [121]. The role of human resource policies is key in facilitating such an attitude change within organisations [121]. Awareness raising and training, obviously, are needed to that end as well [122], as are inclusive attitudes [123:75], and availability of transparent and accessible information as one of the crucial entry points to agency and meaningful participation [124:69].

An inclusive approach to the employment of persons with disabilities entails, likewise, that a siloed approach merely looking into, for example, the positioning of labour legislation vis-à-vis disability is not sufficient, but a more *holistic* approach is needed to see the agency and employment of persons with disabilities as a question cutting across different policy fields, including disability services [73:29]. In this regard it should be noted that the suggested changes regarding working opportunities in the proposed legislation on disability services have been met with some reservations. In the proposal, the day activities (*päivätoiminta*) organised in day centres for some persons with disabilities under the disability service legislation are suggested as alternatives to work related activities and services that support employment to be provided for under the Social Welfare Act [30:214]. Such a categorical approach (which would not give a subjective right to work related activities for all persons with disabilities) has been found to support poorly the varied and changing support needs of persons in terms of their agency in relation to employment, which in practice could benefit from a parallel and flexible use of the different service forms under the two laws [104].

Central in such a holistic approach to enhancing the agency of persons with disabilities in and through employment would also be the recognition of their structural inequality as employees in the labour market. Where disability and vulnerability are understood as socially constructed, the fact that societal structures, including legislation, do not fully recognise persons with disabilities as workforce becomes a source for disability and vulnerability. This is reflected, as Urhonen and Rautianen note, for example in the fact that in Finland the legal requirements on accessibility do not extend to places of work, implying an expectation that persons with disabilities do not work [113]. The first step, therefore, in recognising persons with disabilities as active agents within the labour market would be to acknowledge such sources to inequality as discrimination that is prohibited under law [113, 114]. This, it is argued, is a part of recognising the call for a move away “from identifying jobs that persons with disabilities can or cannot do”, towards hearing their requirements for an equal access to employment [125].

## Conclusion

This special issue explores the role of the vulnerability concept in the move from the legal representation of the rational, self-standing and autonomous human being to acknowledging the inherent irrationality and dependence embedded in the human life. In this article, it was argued that, where used to single out population groups or individuals due to their embodied characteristics, the vulnerability paradigm can be seen to create binaries both among the persons with disabilities, and between the “vulnerable” persons with disabilities and the rational, self-standing and autonomous human being. To mitigate such binaries, an agency-centred discourse of vulnerability was advocated, one that recognises agency as dynamic and responsive to the societal support structures that surround all of us.

One of the central arguments of the article is that generalised approaches do not suffice to make agency a reality for all persons with disabilities. In addition to the measures aiming at universal design, particular, individualised solutions are needed for agency to be possible for all. This is particularly incumbent when addressing disability, given the extensive diversity of intra-group variations between persons with disabilities [23:72]. To overcome objectification and de-agencification – and to enhance agency – this diversity of situations, needs and contexts of lived-in realities of individuals also needs to be expressly reflected in the language, and communication that is used in relation to persons with disabilities. Being intentional in terms of the language is important in this regard [126], as is ensuring that the legal structures holistically support the inclusion of persons with disabilities as active agents in the different areas of their lives. Unfortunately, as was outlined above, this is not always the case in practice. This is visible in how for example in Finland the semiotics of the legal language, and the policies arising therefrom, still often seem to be guided by an ableist paradigm, addressing the agency of persons with disabilities as *a case apart* with a focus on protecting rather than on empowering them as active agents within the society. As a result, such legal structures, together with other multi-layered social mechanisms, tend to sustain the *status quo* where the agency of persons with disabilities and individualised attention to materialise their self-determination rights often is overshadowed by the implicit or explicit protective intent of the law-makers.

In adopting a more inclusive approach to agency, it is important to recognise that agency can take many forms and materialise through different channels. As noted above, collective agency through active disability communities within the civil society can be a powerful tool in advocating for the rights and substantive equality of persons with disabilities. At the same time, individual agency is a necessary precondition for each person to exercise their self-determination rights and to take authority over their lives and the decisions affecting them. Notably, for some individuals, and in different phases of the life-course for all of us, this may materialise in the form of different levels of co-agency, or supported decision-making, which should be recognised as an important tool in realising one’s right to self-determination.

As such, when it comes to the different individualised solutions needed to enable agency for all, more attention in the future research should be attached to the different informal care and support arrangements for co-agency. Such non-formalised structures, and the importance of non-rights, such as love and affection, for the realisation of co-agency and agency [127:198], have often not been sufficiently addressed in the discourse of a human rights-based approach to disability. The Committee on the Rights of Persons with Disabilities reminds in this regard that states parties must recognise the key importance for supported decision-making of such “naturally occurring community support” that takes place for example through family, friends and other social networks [102:para. 45]. Without romanticising the issue, more dialogue is, for example, needed to learn from the experiences for informal care and co-agency in many parts of the Global South where such arrangements often are more deeply and culturally rooted in the societies [127]. At the same time, while such arrangements in practice are an important element in filling in the gaps and compensating for the limits of what states do, the responsibility for ensuring the realisation of agency for all belongs to states as human rights duty-holders. To that end, as aptly noted by Mattson and Giertz “[t]he legal system must be able to take account of different individuals’ way of expressing their will, and yet must be capable too of securing their right to support and protection when their ability to do so fails” [128:157].
